# The interactive dimensions of encounters in HIV care: From trauma to relational traumatic growth

**DOI:** 10.1111/hex.13619

**Published:** 2022-10-01

**Authors:** Jose Catalan, Damien Ridge, Barbara Hedge, Anna Cheshire

**Affiliations:** ^1^ Central North West London NHS Foundation Trust South Kensington and Chelsea Mental Health Centre London UK; ^2^ School of Social Sciences, College of Liberal Arts & Sciences University of Westminster London UK

**Keywords:** death, dying and bereavement, experiencing Illness and narratives, HIV/AIDS, patient–physician relationship, posttraumatic growth

## Abstract

**Introduction:**

A person‐centred model of care, developed in the early days of the HIV epidemic when there were no effective treatments for HIV, led to relatively close relationships between carers and people living with HIV (PLWH). Our study examines the experiences of carers using a relational framework, exploring the traumas and challenges involved, coping practices instigated by carers and the emergence of ‘relational traumatic growth’ opportunities.

**Methods:**

Twenty‐two UK healthcare workers and charity volunteers working with PLWH from the early years of the epidemic were recruited. Semistructured interviews were used to elicit participants' own stories of working with PLWH, from their initial involvement to the present time, and their reflections on the personal impact of working in the field of HIV. Data were analysed using a thematic approach employing relational categories.

**Results:**

The impact of care was related to the formation of close relationships, identification with PLWH, high numbers of deaths and the difficulties and challenges encountered relationally. Participants described attempts to cope through informal and formal support, as well as endeavours to manage professional boundaries. Various ways of making sense of experiences were described, ranging from denial to abandoning the HIV field, to intense commitment. For some, traumatic experiences lead to validation, a search for personal meaning and managing the sense of loss with an exploration of further ventures, contributing to the achievement of relational traumatic growth.

**Conclusion:**

The intensity of relationships in HIV work, developed through the emotional and practical work of caring for PLWH, led healthcare workers and volunteers to experience a range of psychological consequences, both negative (including distress and emotional exhaustion) and also positive (such as acquiring a sense of purpose).

**Patient or Public Contribution:**

People living with HIV and those working with them were involved in the initial study conceptualization and design. The second and fourth authors of this paper were professionals working in HIV throughout the pandemic and have led on all aspects of the study. People living with HIV and those working with them additionally guided participant selection by suggesting participants and supporting recruitment. Narrative transcripts were checked and amended (if necessary) by participants. Initial findings were presented at the AIDS impact conference, where PLWH and those working with them attended and feedback on important ideas that helped to prioritize and shape the study findings.

## 1 INTRODUCTION

The psychological impact of caring for people living with HIV (PLWH) has been well documented since the early days of the epidemic, not only for professionals,^1^ but also for volunteer carers.^2^ Generally, these cross‐sectional studies reveal significant levels of stress and burnout, including high levels of emotional exhaustion, depersonalization and low levels of personal accomplishment.^3^, ^4^ Despite recent significant improvements in the prognosis for PLWH, research continues to highlight the negative psychological impact for healthcare workers caring for PLWH,^5^ although positive aspects of caring have also been described.^6^ Prevention and management of work‐related stress in healthcare has received little attention, beyond sensible recommendations, such as reducing heavy workloads, provision of organizational support^7^ and the use of specific therapeutic interventions, such as mindfulness training.^8^

Historically, HIV care provided by healthcare and charity workers in the United Kingdom took a person‐centred approach, whereby ‘the services … respond to [PLWH's] needs and preferences in a humane and holistic way, [and] the person is a participant, not just a beneficiary’.^9^ Such care relationships developed through a consideration of each person's unique illness experience, and their personal needs for intervention and treatment.^10^ The development, progression and changes to person‐centred care in HIV throughout the evolution of the epidemic have been discussed in detail elsewhere.^9^, ^11^ One feature of this model of care, of importance to the current paper, was the close relationships that developed between healthcare workers and those receiving care. Research shows that in the early days of the HIV epidemic, the poor prognosis, coupled with the stigma and discrimination faced by PLWH and those working in the HIV field,^12^ fostered an unusually close emotional relationship between the carers and those cared for.^11^ Such closeness was beneficial to PLWH, but potentially distressing to the healthcare workers themselves, particularly because the poor prognosis for those living with AIDS meant that carers were exposed to a continuous stream of deaths.

What has up until now not been examined in any depth is the affective relationality of HIV caregiving. While the concept of relationality emerged in psychoanalysis and symbolic interactionist thought,^13^ relational ways of interpreting the world are emerging in a wide range of disciplines from psychiatry to philosophy.^14^ Neither focusing on the individual nor the collective, relationality instead highlights our interdependency and the need to focus on relational categories in all of our analyses.^15^ Conceiving of selves are transactional or ‘interactants’, and affect (states of being) as emergent, personal agency becomes ancillary to relations and the way they line up, especially in organizations like the NHS.^13^, ^15^ In this way of thinking, both patients and professionals are ultimately vulnerable beings.^16^ We cannot help but be pulled into affective dramas, such as during times of epidemics, where ‘…we are [in] constant danger of our self being challenged … [of others] not providing the support we expect; or using our relationships to harm us’ (p. 29).^17^

The efforts of health workers to adjust have been documented both in the early days of the epidemic and more recently.^18^, ^19^ In some cases, the traumatic experiences of caring for people living and dying with HIV resulted in positive transformative changes.^20^, ^21^ Our paper explores relational accounts of participant change—at times positive—that followed the challenges and traumas of caring for people with HIV over extended periods of time.

## 2 AIMS

The current study examined the experiences of healthcare workers and charity volunteers caring for PLWH in the United Kingdom starting before the emergence of effective drug regimens. The aims of the study were to, from a relational perspective, identify traumatic experiences of participants and describe efforts used to cope with them, as well as to explore longer‐term consequences within the accounts, including any evidence of positive change and growth.

## 3 METHOD

### 3.1 Design

The study was qualitative, using one‐to‐one interviews to collect personal narratives. Such narratives reveal the essence of everyday life meanings, they encourage us to listen, as well as consider the moral dimensions of human experience.^22^ Narrative accounts provide a unique window into different worlds, enabling people to put into words experiences in relation to their lives, and in ways that can be understood society‐wide.^23^, ^24^ Ethical approval for the study was obtained through University of Westminster ethical procedures. Written consent from each participant was gained, and a sensitivity protocol was in place for any participants who became distressed in interviews, which included the provision of emotional support to them.^25^

### 3.2 Participants

Healthcare workers and charity volunteers involved in providing care for PLWH in the 1980s and into the 2000s were invited to participate. Inclusion criteria included considerable experience (10 years or more) of caring for PLWH and participants being UK‐based. Exclusions involved living outside the United Kingdom or experiencing severe mental illness. Participants were initially recruited using the authors' professional networks and represented expert patients, HIV nurses and doctors and HIV charity workers. As recruitment progressed, we attempted to include participants missing from our original list (e.g., Black and ethnic minority [BME] participants) via snowball sampling (i.e., where initial participants are asked to suggest new participants from their networks), and to ensure the inclusion of emergent issues of relevance to HIV care.^26^ Twenty‐four participants were invited by email to take part in an interview with one of the authors; 22 agreed to participate. Table 1 provides participants' sociodemographics as self‐reported during interviews or obtained later through a follow‐up email. This sample was drawn from a larger study (*n* = 53) investigating the experience of HIV in the United Kingdom from the mid‐1980s to 2020. Participants who had provided care to PLWH were included in this study.

**Table 1:** Participants' demographics, roles in the epidemic and where given, HIV status

	*N* (%)	*N* (%)	*N* (%)
Demographics	Male	Female	Total
	14 (64)	8 (36)	22 (100)
Ethnicity			
White	14	5	19
African/Afro‐Caribbean	0 (0)	1	1
South East Asian/Indian	0 (0)	2	2
HIV status^a^			
Positive	6	0	6
Negative or not reported	9	7	16
Role^b^			
Health professionals	6	8	14
Activist	5	0 (0)	5
Charity worker	3	0 (0)	3
PLWH	6	0 (0)	6

^a^ Self‐reported as most likely.

^b^ Participants may have more than one role.

### 3.3 Procedure

Interviews were conducted between April 2016 and April 2018, at the participant's home (*n* = 8), place of work (*n* = 10) or at the University (*n* = 4), by J. C. (*n* = 8), B. H. (*n* = 8) and A. C. (*n* = 6). J. C. (male, consultant psychiatrist and academic in HIV) and B. J. (female, clinical psychologist in HIV) had worked in the HIV sector since the beginning of the UK epidemic, and D. R. (male, academic) had worked in the field since the 1980s. Thus, three authors had worked in the HIV field, variously developing services with PLWH and their professional carers, carrying out research on HIV, as well as HIV prevention work; while A. C. (female) was a qualitative researcher in long‐term health conditions. This provided ‘insider’/‘outsider’ perspectives, as the authors worked in different areas of health (including HIV prevention, care and research) enabling them to consider both ‘insider’ and ‘outsider’ interpretations of the data. Some participants knew J. C. and B. H. by reputation or professionally, but we ensured that the interviews were with researchers who did not know the participant. Interview guides were constructed using the narrative interviewer approach that encouraged participants to tell the story of their life working with PLWH, starting with when they first heard about HIV, as encouraging participants to ‘tell their story’ provides one of the richest sources of qualitative data.^22^ However, where some participants found this challenging, asking about specific topics was also used. Our interview guide encouraged storytelling with the opening question ‘Can you tell me about the time that you first heard about HIV and where you were working at the time?’ followed by prompts, such as ‘and what happened next?’. Additionally, a list of topics was included in the schedule which could be used if the participant did not spontaneously cover a topic of interest to the study. An example interview guide is available in the Supporting Information. The interviews were recorded and transcribed verbatim by a professional transcription agency that signed a confidentiality agreement. Interviews lasted on average 81 min, with a range of 20–196 min. Transcripts were checked for accuracy, and identifying names and places were removed from the data, and returned to the participants to check for corrections and additional comments before being finalized.

### 3.4 Analysis

Our analysis centred on relational categories (i.e., the connections between people), for example, interactions with patients and coping strategies that involved others. Data were analysed iteratively and inductively,^27^ using a reflexive thematic approach.^28^ This approach allowed us to explore participants' lived experiences and the complex relational dynamics involved in care work in the HIV field. NVivo software was used to explore and organize the data. Initially, J. C. immersed himself in the data relating to the impact of caring, interrogating it for different time periods and by different participant roles, to look for similarities and contrasts. Draft key themes were written up, and subsequently, all authors were involved in debating the themes (e.g., for priority, importance and relevance). The themes were refined as the manuscript underwent multi‐iterations over a period of 13 months until all authors accepted the final version. The importance of the relationality concept as an overarching concept emerged in the final iteration of the agreed manuscript.

## 4 RESULTS

Study findings are presented around the themes shown in Table 2.

**Table 2:** Narrative themes

Themes	Subthemes
Traumatic experiences emerge relationally	Informal and formal support
Efforts to cope with difficult care experiences	Informal and formal support; Management of professional boundaries; Exiting the field
Towards relational growth from trauma	

### 4.1 Traumatic experiences emerge relationally

In the early years of the epidemic, close relations were encouraged between carers and PLWH through the emerging patient‐centred care model. The rejection PLWH often experienced in their daily lives caused by the stigma of HIV, their sexuality or drug use, also promoted bonding within HIV care settings. Due to the demographics of HIV infection, healthcare professionals and patients were often of similar age and backgrounds, for example, both be men who have sex with men, meaning staff identified with those they were caring for.

It was challenging because they were our age in the main … and they were people like us, and they were getting very sick and dying, and that engendered a very strong camaraderie amongst clinicians and patients… (60, Doctor)

As PLWH became severely ill, developed AIDS, and were thus closer to dying, their medical and support needs tended to increase substantially, leading healthcare workers to spend more time with them, encouraging ever stronger attachments. An intense connection at the point of death was common. For example, the nursing staff was sometimes the last intimate connection for dying people.

When somebody died, we made sure there was always somebody with them, and if they didn't have anybody, we would be with them. I have been with dozens of people when they died, holding their hands, and just sitting there with them… (19, Hospice nurse)

However, relationships with patients did not always end at the moment of death, extending into preparing and laying out bodies, the emotional intensity of which could be particularly challenging for staff who had existing relationships with patients.

So [when somebody died and we needed to prepare the body], I don't know if it was harder for gay staff, but some of the guys we knew … I used to hate having to do it, and the sister would say, you've got to do it, or the porters won't take the body. (23, Nurse)

I cannot think of any conditions, where doctors had friends who died with the same condition … that was a real game changer. (26, Doctor)

Not surprisingly, these experiences of repeated losses and of intimacy disrupted by death took their toll not just on the nurses, but also on other people involved in the care of PLWH. What had originally been described as understandable stress, began to acquire more substantial impacts, and in some cases carers experienced severe mental health symptoms.

I think I knew towards the end that I was becoming a bit bonkers … I was getting the early signs of panic and anxiety, and a lot of us at the time got very irrational illness phobias … later on I started having nightmares that my lungs were full of KS … I had a week off and then did the outpatient clinic because I knew I couldn't do it anymore on the wards. (23, Ward nurse)

A common experience in the early days was the involvement of charity workers, who as volunteers, provided support in the community and on the wards. For them, many of whom were PLWH themselves, the dual task of dealing with their own health concerns while giving support to others could especially evoke trauma.

Those issues were incredibly stressful, but it was an issue of survival at the time, so you weren't able to sit back and think, well you know I am feeling a bit stressed today, so I am just going to just sort of see what I can do, because next week it might be you in that bed. So, it was all hands‐on deck, and that was intensely stressful … it is not just a simple form of PTSD, it's a very complex form. (50, Charity volunteer)

For many, the traumatic consequences of caring for PLWH endured and were still evident, years later, at the time of their interview. Participants reported depression, mood swings and/or posttraumatic stress, where grief could burst through in unexpected circumstances.

I can remember when I'd be driving through the countryside and songs would come on the radio and I'd have to pull over and bawl my eyes out, and then pull myself together kind of thing, but I think it probably did me good crying like that as well, because I didn't do it at the time… (25, Charity worker)

### 4.2 Efforts to cope with difficult care experiences

#### 4.2.1 Informal and formal support

The intensity of caring for large numbers of people who were very ill, including the development of close relationships with patients (many of which would end in loss caused by HIV), required the adoption of strategies to cope with emotions that emerged relationally. Support from colleagues within a team, whether informal or structured, was one way of processing distress. Informal support, simply talking and describing their experiences to each other while sometimes using distancing strategies (e.g., humour), was frequently mentioned.

…there was a lot of humour, often very dark humour as well … I went through a phase where I felt guilty about that dark humour, but it was a survival mechanism. (42, Nurse)

Informal support emergent from relations appeared to be facilitated by the closeness that formed not only with patients but between carers. Such informality was important, as it avoided particular healthcare workers being singled out as being especially affected by trauma and requiring formal support.

Regular formal support group meetings were frequently mentioned by participants. Interestingly, it was nurses, as opposed to doctors, who reported accessing such support groups. A combination of gender (most nurses being female, while most doctors were male at the time), professional expectations (doctors being less willing to see themselves as in need of support) and nurses dealing more directly with the dying, may have contributed to this pattern.

The ward‐based nursing staff were much more comfortable meeting regularly with a psychiatric nurse to discuss their feelings and experiences … they were dealing with people who were dying, and their work was very intense and distressing … it was easier for [medics] to answer the bleep and leave the ward, or do something else, avoiding some of the intensity of distress and discomfort the nurses experienced, and they resisted attempts to provide group support or join the nurses. (51, Psychiatrist)

However, doctors too discovered ways of dealing with emergent difficult feelings, such as via socializing with colleagues at conferences. However, conferences could potentially add to blurred boundaries as the prevailing person‐centred care paradigm encouraged PLWH (who might also be patients of the professional attendees) to attend conferences.

The history of medical conferences was remarkable, where people let their hair down … it was like being off duty, but we were there for a purpose, and with a kind of work hard, play hard ethic … we were a vibrant group who didn't miss a chance to have another beer and talk, and quite soon there were patients coming to meetings. (10, Doctor)

Acknowledging that healthcare workers might themselves require support, in effect meaning they were potentially patients too, was not something that all participants felt comfortable with. Some nurse managers appreciated the delicacy with which formal support for health‐carers had to be offered, and introduced creative solutions, including physical therapies:

We were exploring different ways of supporting nurses … the social worker had a friend who did shiatsu massage, and we had some money, so we bought every nurse a shiatsu massage session, and it was fantastic … and I managed to get it funded on the NHS for a year. (29, Senior nurse)

There was an interesting paradox, as some carers believed that encouragement to open up about trauma needed to be moderated to avoid carers becoming overly focused on their own problems.

I went to counselling sessions, and support groups, and I was learning so much about counselling, its methods and techniques, and making sure you had ways of unburdening … and I talked and talked and shared and shared … but there was a time when I overdosed. (30, Volunteer)

#### 4.2.2 Management of professional boundaries

Managing professional and lay boundaries between healthcare workers and PLWH was not straightforward. While closeness between professionals and patients was understandable and had positive therapeutic consequences, it could also lead to over‐identification, blurring of boundaries and distress when inevitable losses occurred. However, there were differences in approach between those involved in ongoing hands‐on relational work, and those more able to create distance by the nature of their work:

(Compared to general medical wards) it was a world of difference … we would hug patients, people used to go drinking with patients … in hindsight, that wasn't probably a good idea … I think we never called them patients; they were people and sometimes they became our friends. And I think that had a tough emotional impact on us, but I cannot see any other way it could have been. (57, Ward nurse)

I was struck by how involved with the patients the nurses in the medical team were … our mental health team was much more aware of professional boundaries … I think [the nurses] felt we were uncaring because we kept a distance … rather than just jump in and hug patients, we would try to understand what was going on… (51, Psychiatrist)

Due to the potential for blurred boundaries involved in the ongoing care for PLWH, some professionals sought to cope by managing their personal boundaries—creating a physical and emotional distance between patients and their own private lives. For example, having a supportive partner who was not involved in HIV services, or a social life away from work, were considered important distancing measures.

I had a small group of close friends who weren't connected to the charity … who were more than capable of saying, hang on a minute, you've been talking about that too much, now there is a big world out there. (22, Charity worker)

#### 4.2.3 Exiting the field

Reflecting on their experiences over the years, some participants described how living with the relational trauma of constantly losing people they had bonded with, with inadequate support for their own mental health, led them to a critical point where they believed they had to leave the HIV field. It was a similar situation for some who had spent many years as volunteers dealing directly with extended exposure to death and loss.

In the end, I know I coped badly, actually, and I am sure the reason I retired was that I was burnt out, no doubt about it, I was overworked. I got to the stage where I knew, and my own psychiatrist said to me, if you keep doing this, you will be depressed permanently. (33, Doctor)

After 10 years I felt I had done all that I could do, and I didn't necessarily want to do that for the rest of my life, and that time was up. I was exhausted after 10 years and ready … I think the whole thing did take its toll. (52, Charity volunteer)

### 4.3 Towards relational growth from trauma

The impact of loss, fear and trauma—as well as the build‐up of more mundane but additionally dispiriting experiences—was not always acknowledged or addressed at the time, but was frequently recognized by participants sometime later, including during our interviews. The tension between the traumatic experiences, and the need to find ways to survive, and find a better work‐life balance, could deeply and negatively affect participants, including in the ways selves were shaped:

The death thing really gutted me and obviously changed me completely. I don't know what I would have been like if I hadn't been close to those things … I am sure it fucks you up a bit … and obviously it has had an impact. I am not the sort of person who picks at those things though, I tend to lock it away in a box and it stays there, and I am sure the box is quite full, but the lock is holding. (25, charity worker)

Selves were also fashioned from relations at the time in more positive ways, including validation and challenges to internalized homophobia. Witnessing vivid examples of affection and devotion between PLWH and their partners and relatives, had a powerful personal effect, offering a striking contrast with negative and stigmatizing media reports.

I remember this guy … he would slowly clean his partner's mouth, like he was made of porcelain, and he'd ask me, just gently hold his head, just gently, and I will wash it … and seeing such a big guy doing it had more of an impact. And he wouldn't leave this man, he wouldn't even go to the toilet and leave him. And of course, that message, I didn't think about it until recently, meant that being gay wasn't wrong. I knew gay people could love each other, (but) I'd never seen love like it. It moves me to tears thinking about it now, so that was a very positive message to give a young man who was a bit closeted. (23, Ward nurse)

Emergent new directions in life were referred to by several participants. The unique nature and magnitude of the crisis, combined with the personal involvement of healthcare and charity workers, not only sparked feelings of privilege, but also new meanings in life, mentioned repeatedly by participants. For some, gaining life meaning and a purpose was itself a tonic for trauma.

Most of us there felt very privileged to work there, and many people working there were either HIV positive themselves or living with people with HIV, most of the staff were gay … it was a very affected community … initially pretty much everybody had a real investment in it, a real personal investment in it. (19, Hospice nurse)

Trauma and stress were what people were experiencing, and some were saying, this is absolutely terrible but actually it's the best thing that has happened to me in a curious and weird way. I now look after my health, I am surrounded by people who love me, I've got a mission in life. (15, Ward nurse)

The legacy of the affective relations developed in the early epidemic translated into more person‐centred services, according to participants. Reflecting on the needs of PLWH allowed healthcare workers to broaden the scope of their interventions, and to develop services more responsive to the wider emotional needs of their patients.

That's one thing that has changed a lot, so we have a psychosexual therapist, we have psychologists, we have counsellors, we have support groups … we have a group for people who are having difficulty balancing their religion with their sexuality … all those politics from that time have empowered us, I really think that it changed things. (16, psychiatrist)

Eventually, the effectiveness of combination antiretroviral therapy^29^ led to major advances in the treatment of HIV infection, with a significant reduction in the rates of morbidity and mortality. Thus, the need for inpatient care and other services gradually diminished over time from 1996. The significantly improved prognosis for PLWH evoked mixed reactions: delight and relief that people were not dying, but also a sense of loss of role, and a search for new ventures either within HIV charities and services or in other non‐HIV areas. An emergent sense of empowerment seemed to support initiatives to do so.

…there was the sheer growth in the number of people with HIV when people stopped dying … and that created twin challenges of needing to do more on prevention because you had a growing number of people living with HIV and growing pressure on services that had become, crucially, underfunded because there was a decline in public visibility and engagement with HIV issues … we had been dealing with a crisis, by this point, for 15, 20 years … you cannot live in crisis for too long when you have already been in crisis for a very, very long time. So, just as a number of volunteers had moved on, others had found other concerns, new ways of working. (22, Charity worker)

## 5 DISCUSSION

We examined accounts and reflections of the healthcare workers, and charity volunteers regarding their experiences of supporting PLWH who were seriously ill and dying, taking an epistemologically relational approach.^13^ We documented unusually close affective relationships between PLWH and their healthcare workers/support providers. By foregrounding relations as a lived reality, we were able to elucidate how valued meanings and even life purpose were emergent from the connections participants described.^30^, ^31^ This was evident in many of our findings, such as when participants highlighted the intimacy involved in the rituals around death; the ways in which closeness (including friendships) was forged with patients; how participants talked about needing to find ways to strategically distance themselves from patients; as well as the way conferences became ways to let off steam, connect with others and engage in a kind of collective therapy. There were highly challenging aspects to the way relations developed, like common experiences of trauma in losing people who had become emotionally close, with some people believing that they had to leave their roles to cope. There were blurred personal boundaries, which could make it difficult for participants to switch off and get the downtime they needed. Consistent with other relational analyses pertaining to health,^32^ identities and selves themselves were transactional and emergent too, such as when people believed they were changed by the trauma, for better (e.g., challenging internalized homophobia) or for worse (e.g., ‘it fucks you up a bit’). Relations with PLWH had given some participants a sense of empowerment, helping to validate their own feelings, for example about their sexuality, or strengthening their sense of agency and ability to make an impact in response to their patients' needs. For some, it meant gaining a ‘mission’ in life. Importantly, gaining meaning or purpose in life could be a way to manage trauma. Many times, carers and managers had to find clever ways of relating to others to enact self‐care and survive, for example, via creating some distance with patients despite the intimacy involved, or providing informal support to avoid stigmatizing carers with labels.

These relational findings emerged against the distinctive backdrop of HIV care, which included carers being strongly drawn to the area by a sense of responsibility towards stigmatized groups, as well as the challenge and novelty of the disease. Additionally, carers were often similar in age to those they cared for, not to mention sexual orientation, with many also having friends with HIV. PLWH were themselves involved in helping professionals to understand how to treat the illness. However, the reality of participant experiences was that HIV encounters could take their toll. For most, it led to a strengthening of their commitment, and endeavours to make the relational dimensions of the work safer. Narratives were imbued with tales of stressful and traumatic encounters, efforts to cope with such experiences, and in some cases, the development of a kind of ‘relational traumatic growth’ as outlined in this paper.

In the early years of the epidemic when there were no effective treatments for HIV, healthcare workers' experiences were mostly centred on unrelenting suffering and death of PLWH. The accounts of our participants are consistent with previous reports of physicians and activists describing their efforts to cope with the unprecedented crisis.^33^ Our research also aligns well with the results of research studying burnout and the impact on healthcare workers of dealing with large numbers of deaths amongst people being looked after.^1^, ^34^ Bayer et al.^19^ have documented the profound impact of what they call the early ‘dark years’ of AIDS on doctors, where avant‐garde approaches (e.g., unconventional therapies) and collegiality became important. Exposure to—and involvement in—the deaths of many PLWH was faced up to.^35^ These experiences took their toll, with some participants describing work‐related anxiety and panic attacks, as well as symptoms of posttraumatic stress disorder.

In sum, while emotional experiences were individually experienced, they were socially emergent in a myriad of ways in HIV care situations, for better or for worse.^36^ Thus, there was some predictability to the issues that emerged for people in HIV care, revolving around common issues like (over) identification, burnout, grief, trauma and more positively in terms of inspiring meaningful life experiences and choices. Jacob and Lagace‐Roy^20^ have documented how the personal and professional identities of gay physicians changed in response to the stresses of caring for people with HIV/AIDS over almost three decades. Transformations included increased militancy, a move towards viewing patients as equals and a reduction in therapeutic distance.

There were some reported differences in preferences for support between professional groups, with nurses (mainly female) being in general more interested in joining ward‐based support groups than doctors (who were mainly male). Managing professional boundaries was an important aspect of the participants' efforts to deal with the stressful circumstances they were facing. The similarity in age and background (including sexuality), added to the distressing experience of recognizing an approaching death, making it easy for healthcare workers to identify with—and become attached to—the PLWH they were looking after. While such intimate professional relationships contributed in general to good quality personalized care, it also placed greater pressure on the psychological resources of healthcare workers to maintain some degree of emotional separation, both to ensure objectivity in terms of the care provided, and to maintain their own ability to see themselves as professionals and not just friends of the patients, as described by Bayer et al.^19^ Other efforts to manage and maintain emotional distance included taking breaks from work and having a personal life outside their role as HIV professionals, and if everything else failed, leaving the field of HIV care.

The concept of posttraumatic growth (PTG) has received a good deal of attention in recent years.^37^ There has been only limited research on PTG in relation to HIV infection, and what has been published concerns people living with HIV, rather than the healthcare workers as we have described in this paper.^38^, ^39^, ^40^, ^41^ In the conceptualization of PTG proposed by Tedeschi and Calhoun,^37^ several domains are identified, including a greater appreciation of life and a changed sense of priorities; warmer and more intimate relationships with others; a greater sense of personal strength; a recognition of new paths for one's life; as well as spiritual development. Many of these points were echoed in the relational analysis of accounts given by our participants. Several felt privileged for having had the opportunity to work in HIV care and to have provided services to PLWH, especially during the early days of the historic epidemic. Their feelings of privilege were linked to a sense of direction and commitment, and to acquiring meaning and purpose in life as a result of their work, even when such positive feelings were clouded by difficult memories. A sense of personal validation in their work was described by other participants. In their study of gay physicians involved in HIV care, Jacob and Lagace‐Roy^20^ report positive transformative changes in both the personal and professional identities of their participants as a result of their experiences. However, changes may not always be of a positive nature, such as when healthcare workers who had worked hard to provide good care and develop services, later found themselves no longer to be in demand.^42^ In a reversal of the acquisition of meaning at the time of crisis reported by some participants, some now found their lives had lost meaning and direction. The contribution of our paper is that the focus in our narratives was on the relational dimensions of traumatic growth, which the concept of PTG side‐lines.

In terms of limitations, we cannot comment on what the impact of caring for PLWH would have been had a more traditional form of care been maintained, rather than a person‐centred care approach. Perhaps such an approach, while being clinically efficient, might have led to a greater emotional distance between carers and patients, possibly reducing the adverse psychological consequences for carers. As the researchers facilitated the recruitment of participants through their personal and professional contacts, this may have restricted the range of opinions reported. However, two of the researchers (A. C. and D. R.) were not part of the UK history of HIV, so they were able to suggest participants, interrogate the data and conduct analysis as relative outsiders. As these narratives were collected from a specific healthcare system (i.e., NHS), other settings may yield different perspectives. Additionally, workers who left the field sooner than our long‐standing workers may also have alternative narratives. Our findings are likely applicable to other health areas. As the world faces a new and intense pandemic, there is already evidence of adverse mental health consequences of caring for COVID‐19 patients.^43^, ^44^, ^45^ As back then, there are some suggestions that many COVID workers prefer informal and organizational support over formalized professional help.^46^ Our work to understand the factors that could contribute to reducing the impacts of such care could be instructional for the new pandemic, where there are similar features, for example, a high caseload of death, relational intensity, lack of community understanding about what COVID care entails, stigma and misinformation. Our study highlighted the difficulties to be expected in long‐term intimate person‐centred care and suggests how carers in current or future pandemics might be better supported to provide good quality care, whilst caring for themselves and avoiding burnout.

## Supporting information

Supplementary information.

## Data Availability

I note the data collected may be retained in an anonymized archive and I am happy for my data to be reused as part of future research activities.
