# Supplementary material for: The interactive dimensions of encounters in HIV care: From trauma to relational traumatic growth
Source: Health Expect. 2022 Oct 1;25(6):3114–23. doi: 10.1111/hex.13619 (PMC9700152; doi:10.1111/hex.13619)
Supplement: Supplementary file 1 — Supplementary information. [file HEX-25--s001.docx]

**CHANGES IN THE CARE & SUPPORT FOR PWHIV**

**INTERVIEW SCHEDULE – HEALTH PROFESSIONALS**

**INTRODUCTION**

- Thank participant for taking part.
- Introduce self and the project.
- Then provide information about the interview:
  - I am interested in your stories about the care and support for PWHIV/AIDS in the UK, from the beginning of the epidemic until the present time, focusing on what the NHS, communities, social services, and voluntary organizations, provided (whether they be good, lacking, novel or just terrible!).
  - I would like to know about your experience and/or that of your patients and colleagues. There are no right or wrong answers, we just want to build a picture of what it has been like from your point of view.
  - Say how long it will take, check how long they have, remind them they can take break if they want, finish the interview on another day.
  - I would like to record the interview, and unless you are happy for your name to be identified, it will be treated in complete confidence.
- Any questions?
- Check participant is happy to start

**INTERVIEW GUIDE**

**Part A - Getting the narrative**

*Opening question*

I’m here today to get your ‘story’ about your experiences of providing care and support to people with HIV.

Can you tell me about the time that you first heard about HIV and where you were working at the time?

*Topics to cover*

How did you come to start working in the HIV field?

Prompts (all bulleted questions are prompts to encourage the participant to talk further on the topic, to be used if needed)

- What motivated you to work in this area?
- What was it like when you first starting working in the HIV field?
- What treatment options were available to patients?
- What kind of care were you providing for patients?
- What was it like working with HIV patients? (wards, outpatients, charities etc.)
- What were the challenges working in this field?
- What were the rewards of working in this field?
- What was it like dealing with death and dying?
- What were your impressions about the patient experience at this time? What your relationship with the patients like?
- Did you suffer any stigma or other problems from working in this field?

What other health professionals/agencies were you working with at this time?

- What was your relationship with other health professionals/agencies like?
- What was the psychological support provided to patients like?

How did the treatment/care you were providing change after your initial years working in HIV, if at all?

- Did your job role changes
- Were you trying any new treatments/ways of caring for patients?

If you were working in the 1990s, what impact did the introduction of combination therapy have on your work?

- Where were you working at that time?
- What effect did the introduction of combination therapy have on your patients?
- What were the implications for the treatment you were providing?

How did the care you were providing change in the years after combination antivirals were introduced (1996)?

If you continued working in the HIV field, what kept you in the field?

Thinking more about the present day, what is the care for PWHIV like now?

- Compared with beginning?
- What are the key challenges in working with PWHIV?
- What is your relationship with other health and social care providers like now?
- If you are working in the field still, how do you feel about working in the field now?
- What has changed about NHS care over the years (better/worse)?
- What do you feel could be improved in relation to current service provision?

**Part B - Additional questions**

Where there any political forces or social movements influencing the care you were providing over the years?

- Political agendas?
- Activism?
- Media?

Have the changing attitudes of society influenced the care you have provided to PWHIV over the years?

Have there been any positive changes to NHS care that have emerged from the HIV epidemic?

What were relationships like between patients and health professionals earlier on? Has this changed over time?

What have been the highlights for you working in this field?

Have you personally experienced any positive/negative effects from working in this field?

- Anything learnt
- Altered perspectives on life
- Effects on relationships

What learning could be transferred over from HIV to apply to care for other long-term conditions?

Is there anything else you want to say, but you have not had a chance to say it?

Notes

Most important is we want to know about medical, community, voluntary and social care, and how this changed over time

If possible, check which time periods they are referring too.
